# Design and Analysis of a New Hair Sensor for Multi-Physical Signal Measurement

**DOI:** 10.3390/s16071056

**Published:** 2016-07-08

**Authors:** Bo Yang, Di Hu, Lei Wu

**Affiliations:** 1 School of Instrument Science and Engineering, Southeast University, Nanjing 210096, China; 220142690@seu.edu.cn (D.H.); 220152695@seu.edu.cn (L.W.); 2 Key Laboratory of Micro-Inertial Instrument and Advanced Navigation Technology, Ministry of Education, Nanjing 210096, China

**Keywords:** hair sensor, acceleration, angular velocity, air flow rate, resonant transducer

## Abstract

A new hair sensor for multi-physical signal measurements, including acceleration, angular velocity and air flow, is presented in this paper. The entire structure consists of a hair post, a torsional frame and a resonant signal transducer. The hair post is utilized to sense and deliver the physical signals of the acceleration and the air flow rate. The physical signals are converted into frequency signals by the resonant transducer. The structure is optimized through finite element analysis. The simulation results demonstrate that the hair sensor has a frequency of 240 Hz in the first mode for the acceleration or the air flow sense, 3115 Hz in the third and fourth modes for the resonant conversion, and 3467 Hz in the fifth and sixth modes for the angular velocity transformation, respectively. All the above frequencies present in a reasonable modal distribution and are separated from interference modes. The input-output analysis of the new hair sensor demonstrates that the scale factor of the acceleration is 12.35 Hz/g, the scale factor of the angular velocity is 0.404 nm/deg/s and the sensitivity of the air flow is 1.075 Hz/(m/s)^2^, which verifies the multifunction sensitive characteristics of the hair sensor. Besides, the structural optimization of the hair post is used to improve the sensitivity of the air flow rate and the acceleration. The analysis results illustrate that the hollow circular hair post can increase the sensitivity of the air flow and the II-shape hair post can increase the sensitivity of the acceleration. Moreover, the thermal analysis confirms the scheme of the frequency difference for the resonant transducer can prominently eliminate the temperature influences on the measurement accuracy. The air flow analysis indicates that the surface area increase of hair post is significantly beneficial for the efficiency improvement of the signal transmission. In summary, the structure of the new hair sensor is proved to be feasible by comprehensive simulation and analysis.

## 1. Introduction

Micro-autonomous systems (MAS) that are autonomous, multifunctional, collaborative ensembles of agile, mobile microsystems are developed to enhance tactical situational awareness in urban and complex terrains for small unit operations. The sensory systems in the MAS need to realize dynamic robot control, mapping and navigation, situational awareness and so on. However, the very limited payload capacity in volume, weight and power restricts the application range of current commercial sensors in the MAS or no commercial parts are available even if the constraints in size and weight are relaxed. The biomimetic hair sensors with a variety of sensing capabilities, high volumetric efficiency and high sensitivity are expected to be potentially applicable in MAS. Multiple biomimetic hair sensors to detect a variety of physical signals were studied in the last twenty years [1,2,3]. References [4,5] developed a hair sensor with a micro-hydraulic amplification system and a single capacitance signal transducer for air flow measurement. Air flow resolution down to 2 cm/s was achieved through a 4-cell array device. Biomimetic hair-flow sensors based on a differential capacitance signal transducer were presented in [6,7]. A biologically inspired fish lateral line sensor which is a micromachined, distributed flow sensor based on the piezoresistive effect was reported in [8,9]. Inspired by the principles of insect bionics, many institutes have researched hair biomimetic micro-inertial sensors in order to meet the application requirements in insect-scale MAS [10]. Several groups have successfully developed biomimetic hair acceleration sensors with a differential capacitive transformation [11,12]. Simultaneously, a bionic micro-gyroscope with the piezoelectric drive was researched for the motion control of future mm scale micro aerial vehicle (MAV) [13]. However, most currently developed hair sensors have some significant disadvantages, including relatively simple functions, larger volume and lower sensitivity in the signal transducer, which makes it difficult to meet the future demands of MAS. This paper presents the design and analysis of a new hair sensor for multi-physical signal measurements. Three physical signal detection functions, including acceleration, angular velocity and air flow, are integrated into a single device with a high precision resonant signal transducer. The description and design for the device are given in Section 2; Section 3 presents the modal simulation; Section 4 illustrates the performance analysis; concluding remarks are finally given in the last section.

## 2. Device Description and Design

**Figure 1:**
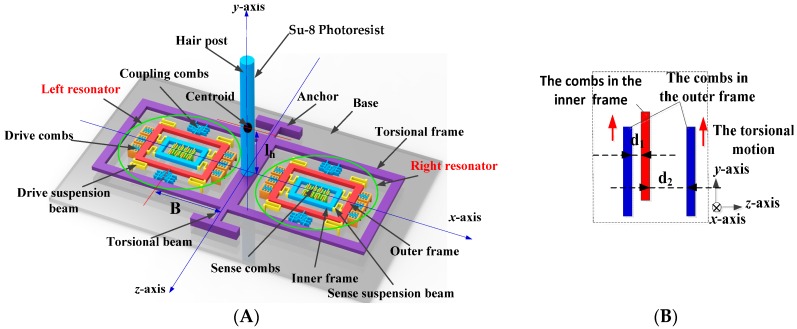
The structure scheme of hair sensor. (**A**) The whole structure; (**B**) The coupling combs.

The new hair sensor features three signal detecting capabilities, including the air flow rate, the acceleration and the angular velocity, which can be used for motion control and situational awareness in a MAS. The integration in three kinds of sensing function in the new hair sensor helps to further reduce the size, decrease the weight and lower the power consumption. The structure scheme of the new hair sensor for multi-physical signal measurement is shown in Figure 1A. The entire structure consists of two main parts: the hair post and the signal transducer. The hair post is mainly used to interact with the physical signal (air flow rate and acceleration) and transmit the physical signal to the signal transducer. The signal transducer is used to convert and extract the signal. The signal transducer is constituted of a torsional frame and two symmetrical resonators. The torsional frame is not directly connected to two resonators, but rather is coupled with the above two resonators by the coupling combs. The structure parameters are shown in Table 1.

### 2.1. Air Flow Measurement

Firstly, the left and right resonators are driven by the drive combs along the *z*-axis. The drive signal is extracted and fed back to the drive combs by external control circuits. Then the closed-loop self-excitation circuits are locked to the natural frequencies of the resonators. When the air flow rate is input along the *x*-axis, the hair post is promoted to rotate along the *x*-axis by the drag force. The rotation movement is transferred to the torsional frame and is eventually converted into an approximately linear motion along the *y*-axis in the coupling combs, shown in Figure 1B. The overlap area of coupling combs may vary due to the input of air flow rate. When a bias voltage V is applied on the coupling combs, an electrostatic force and a negative electrostatic stiffness are exerted on the resonator along the *z*-axis. The change of the overlap area in the coupling combs results in the variation of the negative electrostatic stiffness, which will alter the effective stiffness or the natural frequency of resonators along the *z*-axis. Therefore, the input of air flow rate can be measured by detecting the frequency variation of the closed-loop self-excitation system.

Suppose the drag force exerted on the hair post is estimated by [9]:

(1) $$F_{f}\approx \int_{0}^{L_{H}}\frac{1}{2}C_{D}(\mu_{i})\rho D_{i}u_{i}^{2}dz$$

where *u_i_* is the local flow velocity of the hair post, *C_D_*(*u_i_*) is the local drag coefficient, *ρ* is the fluid density, and *D_i_* is the local diameter of the hair post, *L_H_* is the length of the hair post.

Integrating local drag force over the length of the hair post will give us an estimate of the moment acting at the base of the hair post:

(2) $$M_{f}=\int_{0}^{L_{H}}\frac{1}{2}C_{D}(\mu_{i})\rho D_{i}u_{i}^{2}zdz$$

Suppose the hair post diameter, *D_i_* and the local flow velocity, *u_i_* are approximately constant, and ignore the differences of drag coefficients in different flow velocities, the moment can be simplified as:

(3) $$M_{f}=\frac{1}{4}C_{D}\rho Du^{2}{L_{H}}^{2}$$

where *D* is diameter of hair post, *u* is the flow velocity. The moment is related with not only the hair surface area, *DL_H_* but also hair length, *L_H_*.

The hair post and the torsional frame can be simplified as a spring-mass-damper system [14]:

(4) $$J\frac{d^{2}\theta }{dt^{2}}+b\frac{d\theta }{dt}+k_{o}\theta =M$$

where:

$$J=J_{1}+\frac{\pi \rho D^{2}}{48}(4{L_{H}}^{3}+\frac{3}{4}D^{2}L_{H}),\;\omega_{T}=\sqrt{\frac{k_{o}}{J}}$$

and *J* is the total moment of inertia, *J*_1_ is the inertia moment of the torsional frame and the remaining part is the inertia moment of hair post, *ω_T_* is the resonant frequency of torsional frame, *b* is the viscous damping coefficient, *k_o_* is the torsional stiffness, *M* is the input moment, *θ* is the angular displacement.

The angular displacement, *θ* is measured by converting it into a frequency. According to the Equations (6) and (7) (shown in Section 2.2), the mechanical sensitivity of air flow rate is:

(5) $$S_{f}=\frac{df}{du^{2}}\approx \frac{n\varepsilon LV^{2}BC_{D}\rho DL_{H}^{2}}{16\pi^{2}f_{0}d_{1}^{3}m_{r}k_{o}}$$

The frequency variation is approximately linear with the square of flow velocity.

### 2.2. Resonant Signal Transducer

The torsional movement is converted into a frequency variation by the resonator and the electrostatic coupling combs [15]. The relationship between the moment and the frequency is:

(6) $$f\approx f_{0}+SM$$

where *S* is the sensitivity coefficient from the moment to the frequency and:

(7) $$S=\frac{n\varepsilon LV^{2}B}{4\pi^{2}f_{0}d_{1}^{3}m_{r}k_{o}}$$

where *n* is the number of coupling combs, *L* is the length of coupling combs, *V* is the bias voltage applied on the coupling combs, *B* is the equivalent distance from the electrostatic coupling combs to the *z*-axis (shown in Figure 1A), *d*_1_ is the coupling combs gap and *d*_1_ << *d*_2_, *m_r_* is the proof mass of resonator, *f*_0_ is the static frequency of the resonator and:

(8) $$f_{0}=\frac{1}{2\pi }\sqrt{\frac{\left(k_{r}-\frac{2n\varepsilon LV^{2}H}{d_{1}^{3}}\right)}{m_{r}}}$$

where *k_r_* is the stiffness of resonator and *H* is the comb thickness. The sensitivity coefficient can be increased by decreasing the static frequency *f*_0_ and coupling combs gap *d* or adding a bias voltage *V*.

### 2.3. Acceleration Measurement

Similarly, when the acceleration is input along the *x*-axis, the hair post and the torsional frame are promoted to rotate around the *z*-axis by the inertial force. Suppose the inertial moment exerted on the hair post is estimated by:

(9) $$M_{a}=\int_{0}^{L_{H}}\rho_{H}\pi {\left(\frac{D}{2}\right)}^{2}a(t)zdz=m_{h}l_{h}a(t)$$

where:

$$m_{h}=\frac{\pi \rho_{H}D^{2}L_{H}}{4},\;l_{h}=\frac{L_{H}}{2}$$

and *m_h_* is the proof mass of hair post, *l_h_* is the length from the centroid to the base, *α*(*t*) is the input inertial acceleration, *ρ_H_* is the density of hair post. The moment is related with not only the hair proof mass *m_h_* but also hair centroid *l_h_*.

According to Equations (6) and (7), the mechanical sensitivity of acceleration is:

(10) $$S_{a}=\frac{n\varepsilon LV^{2}Bm_{h}l_{h}}{4\pi^{2}f_{0}d_{1}^{3}m_{r}k_{o}}$$

By taking the mechanical sensitivity and bandwidth, a figure of merit (FM) can be defined for the hair accelerometer, which is similar to the approach describe by [16]:

(11) $$FM=sensitivity\times bandwidth=\frac{n\varepsilon LV^{2}Bm_{h}l_{h}}{4\pi^{2}f_{0}d_{1}^{3}m_{r}k_{o}}\times \sqrt{\frac{k_{o}}{J}}=\frac{n\varepsilon LV^{2}Bm_{h}l_{h}}{4\pi^{2}f_{0}d_{1}^{3}m_{r}}\sqrt{\frac{1}{k_{o}J}}$$

$$FM\approx K\sqrt{\frac{D^{2}L_{H}}{k_{o}}}$$

where $K=\frac{n\varepsilon LV^{2}B}{16\pi f_{0}d_{1}^{3}m_{r}}\sqrt{\frac{3\rho_{H}}{\pi }}$. The overall performance merit can be improved by increasing the hair diameter *D* and hair length *L_H_* or decreasing the torsional stiffness of *k_o_* in the condition of compromise between sensitivity and bandwidth.

### 2.4. Angular Velocity Measurement

When the resonator is driven by the electrostatic force, the outer frame vibrates together with the inner frame along the *z*-axis. Suppose the angular velocity is input along the *y*-axis, the inner frame is motivated to vibrate along the *x*-axis by the Coriolis acceleration. Due to the motion restriction in the drive suspension beam along the *x*-axis, the outer frame will almost remain stationary in the *x*-axis. The Coriolis movement in the inner frame can be detected by the sense combs. The sense displacement amplitude is:

(13) $$x=\frac{-2\omega_{d}\Omega z}{\sqrt{{\left(\omega_{nx}^{2}-\omega_{d}^{2}\right)}^{2}+\frac{\omega_{nx}^{2}\omega_{d}^{2}}{Q_{x}^{2}}}}$$

where *ω_d_* is the drive frequency of the resonator along the *z*-axis, *ω_nx_* is natural frequency of sense mode along the *x*-axis, *z* is the drive displacement amplitude, *Q_x_* is the quality factor of sense mode and Ω is the input angular velocity.

The sense displacement is influenced by the frequency variations of the resonator *ω_d_* from the input of acceleration or air flow rate. The changed rate of displacement relative to the resonant frequency is:

(14) $$\frac{dx}{d\omega_{d}}=\frac{-2\Omega z(\omega_{nx}^{4}-{\omega_{d}}^{4})}{{\left({\left(\omega_{nx}^{2}-\omega_{d}^{2}\right)}^{2}+\frac{\omega_{nx}^{2}\omega_{d}^{2}}{Q_{x}^{2}}\right)}^{\frac{3}{2}}}\approx \frac{-\Omega z}{{\left(\omega_{nx}-\omega_{d}\right)}^{2}}$$

The change rate can be decreased by increasing the frequency differences between the drive mode and sense mode.

## 3. Modal Simulation and Optimization

**Figure 2:**
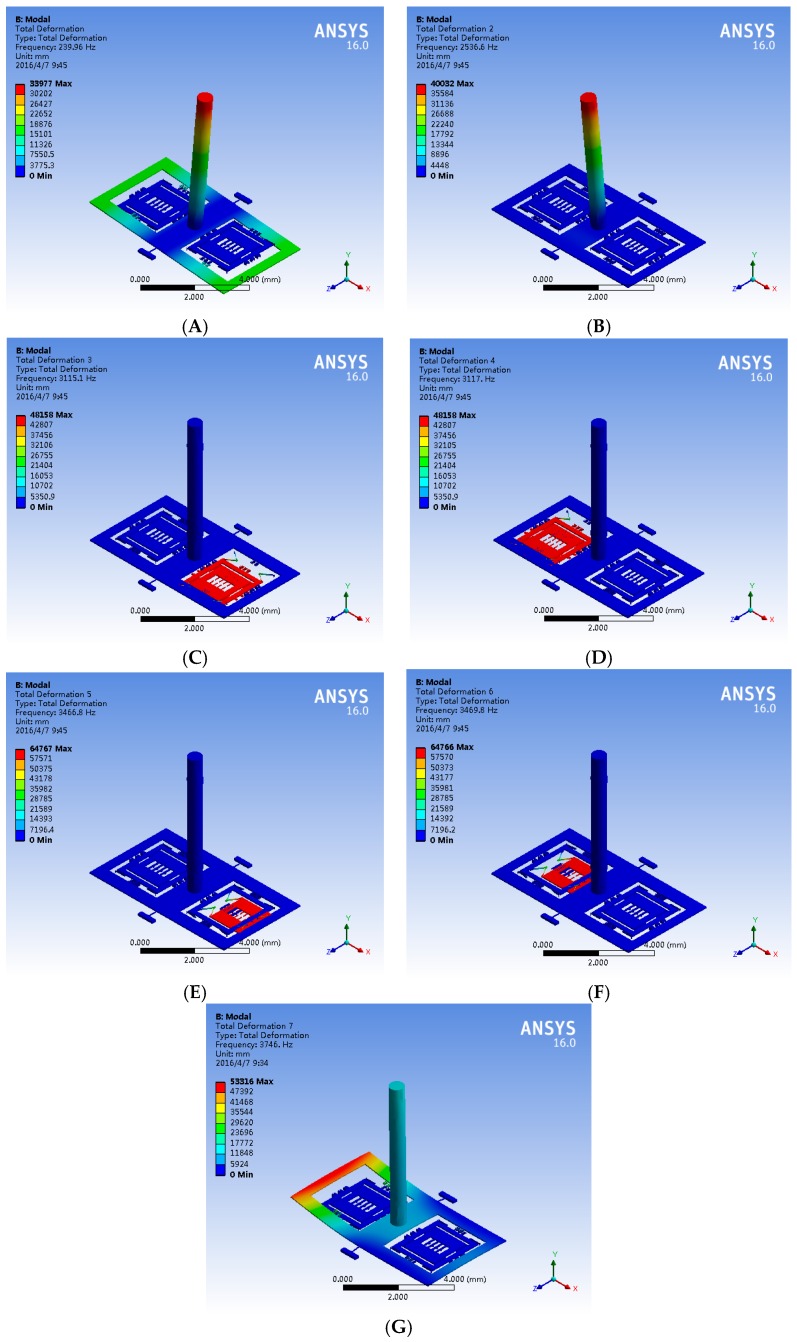
The structure mode of hair sensor. (**A**) The air flow and acceleration sense mode; (**B**) The bending mode of the hair post; (**C**) The resonator mode 1; (**D**) The resonator mode 2; (**E**) The gyroscope sense mode 1; (**F**) The gyroscope sense mode 2; (**G**) The curling mode of torsional frame.

According to the material properties shown in Table 2, a modal simulation is implemented in order to verify the principle of the hair sensor and optimize the modal distribution. The finite element model of the hair sensor is optimized by ANSYS software. The first seven modes shown in Figure 2 are extracted and the first seven mode frequencies of the hair sensor are shown in Table 3.

The first mode shown in Figure 2A is the sense mode of air the flow rate and the acceleration. The mode, which is mainly a torsional movement of the torsional frame driven by the hair post, illustrates the signal conversion from the air flow rate or the acceleration into the torsional movement. Theoretically, the decrease in the mode frequency benefits the improvement in the signal-converting sensitivity. However, too low a frequency of the first mode will result in a narrow bandwidth. The conversions of the air flow rate and the acceleration share the common first mode, but the working mechanisms are completely different. The measurement of air flow rate is related to the surface characteristics of the hair post (such as the surface area) and there must be direct contact with the hair post, while the detection of acceleration is associated with the mass and the mass distribution of hair post in the signal conversion. In practical applications, the measurement of air flow rate and acceleration can be distinguished through the physical isolation, structure shape optimization of the hair post and the array technology. The third and fourth modes shown in Figure 2C,D are the resonant movements of resonators along the *z*-axis. Combined with the negative stiffness effect of the coupling combs, the signal conversion from the torsional movement to the frequency variation is achieved by the resonant mode. According to Equation (7), the decrease in the natural frequency and the proof mass of the resonator can increase the sensitivity of the signal conversion. However, the low resonant frequencies of resonators can be influenced by the vibration and shock. The fifth and sixth modes shown in Figure 2E,F are the sense movement of the inner frame along the *x*-axis. Coupling the third and fourth modes with the Coriolis effect, the angular velocity signal is sensed by the fifth and sixth modes. The second and seventh interference modes shown in Figure 2B,G are the bending modes of the hair post and the torsional frame. The useful modes are isolated obviously from the interference modes. The mode simulation results demonstrate the basic principle of the hair sensor for the multi-physical signal measurement (air flow, acceleration and angular velocity) is feasible.

## 4. Performance Analysis

### 4.1. The Input-Output Characteristics

**Figure 3:**
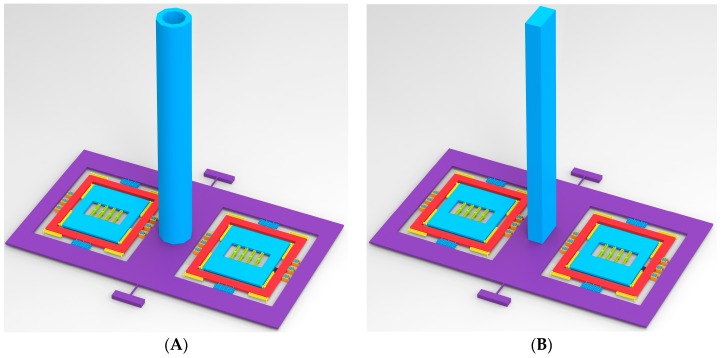
Different surface shapes of hair post for air flow rate sensitive optimization. (**A**) Hollow circular hair post; (**B**) Rectangle hair post.

**Figure 4:**
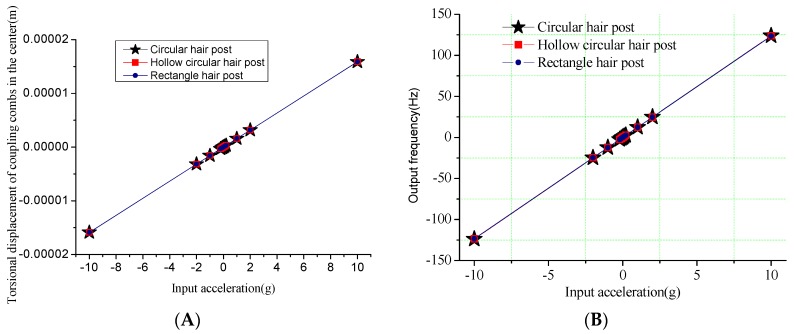
The signal outputs in the input of acceleration along *x*-axis. (**A**) Output torsional displacement versus acceleration; (**B**) Output frequency versus acceleration.

**Figure 5:**
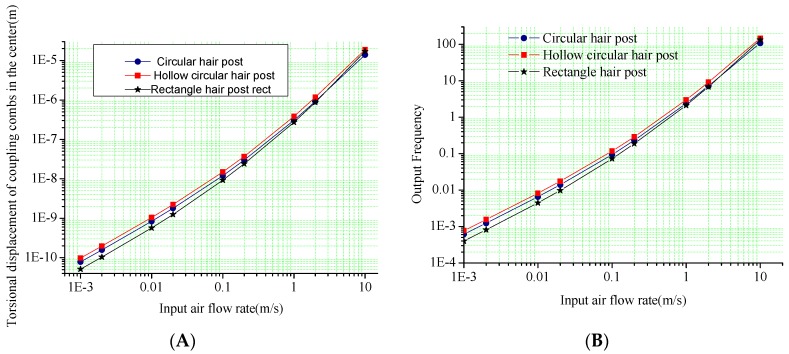
The signal outputs in the input of air flow rate along the *x*-axis. (**A**) Output torsional displacement versus air flow rate; (**B**) Output frequency versus air flow rate.

The input-output relationship is simulated to verify the mechanical characteristics of the hair sensor structural. Firstly, the different surface shapes of hair post shown in Figure 3 are optimized to improve the sensitivity of the air flow rate in addition to the circular structure shown in Figure 2A. Figure 4 and Figure 5 show the outputs of the air flow rate and the acceleration for different surface shapes of the hair post. When the acceleration is input from +10 g to −10 g, the torsional displacements in the center of the coupling combs and frequency variation of the resonators are shown in Figure 4. Structural optimization of hair post does not change sensitivity characteristics of the acceleration. The scale factors of the torsional displacement and the frequency variation shown in Figure 4 are approximately 1.59 μm/g and 12.35 Hz/g, respectively. The simulation results verify that the new hair sensor with a resonant signal converter can measure the acceleration along the *x*-axis. The comparison in the performance of three kinds of hair structures is shown in Table 4. Similarly, when the air flow rate is input from −10 m/s to +10 m/s, the torsional displacements in the center of coupling combs and the frequency variation of the resonators are shown in Figure 5. Apparently, the new hair sensor with different hair posts can measure the air flow rate along the *x*-axis by means of the same resonant conversion principle. Compared with the acceleration detection, the significant difference is that the hollow circular structure of the hair post achieves maximum sensitivity in the measurement of the air flow rate through structural optimization. Simultaneously, compared with the circular hair post, the rectangle hair post has larger sensitivity in the air flow rate region from 1 m/s–10 m/s (the sensitivity of the rectangle hair post is 1.324 Hz/(m/s)^2^, while the sensitivity of circular hair post is 1.059 Hz/(m/s)^2^), which is consistent with conventional fluid knowledge. However, the sensitivity of the rectangle hair post is slightly less than that of the circular hair post in the air flow rate measurement from 1 mm/s to 1 m/s (the sensitivity of the rectangle hair post is 4.640 Hz/(m/s)^2^, while the sensitivity of circular hair post is 5.736 Hz/(m/s)^2^). The possible reason is the bending stiffness of the rectangle hair post is less than that of the circular hair post along the *x*-axis, and the bending of the hair post absorbs part of the air flow rate energy. Additionally, a significant difference of sensitivity can be observed between the high flow rate and the low flow rate range. The main reason is the sensitivity is directly associated with the local drag coefficient C_D_(u_i_) according to the Equation (5). Furthermore, the local drag coefficient C_D_(u_i_) varies depending on the flow rate. The hair sensor can sense approximately the air flow rate of 1 mm/s if the frequency detection circuit has a responsivity of 0.001 Hz.

**Figure 6:**
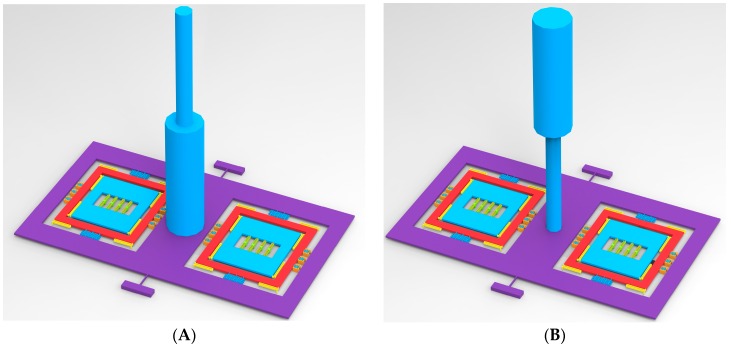
Hair post structures with different center of gravity for acceleration sensitive optimization. (**A**) I-shape hair post; (**B**) II-shape hair post.

**Figure 7:**
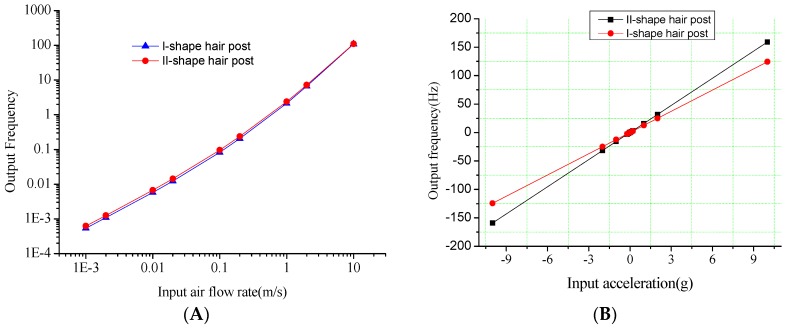
The signal outputs along the *x*-axis in the hair post structures with different centers of gravity. (**A**) Output frequency versus air flow rate; (**B**) Output frequency versus acceleration.

Similarly, the hair post structures with different centers of gravity shown in Figure 6 are optimized for the sensitivity improvement of acceleration. Figure 7 shows the output of the acceleration and the air flow rate in the hair post structures with different centers of gravity. The comparison in the performances of two kinds of hair structures is shown in Table 5. Provided the surface area of the hair post is nearly the same, structural optimization of the hair post fundamentally does not change the sensitivity characteristics of air flow rate. However, the acceleration sensitivities in the different structures of the hair post differ significantly due to the variation in the gravity center. The II-shape structure of the hair post achieves the maximum sensitivity acceleration of 15.89 Hz/g through the optimization of gravity center.

**Figure 8:**
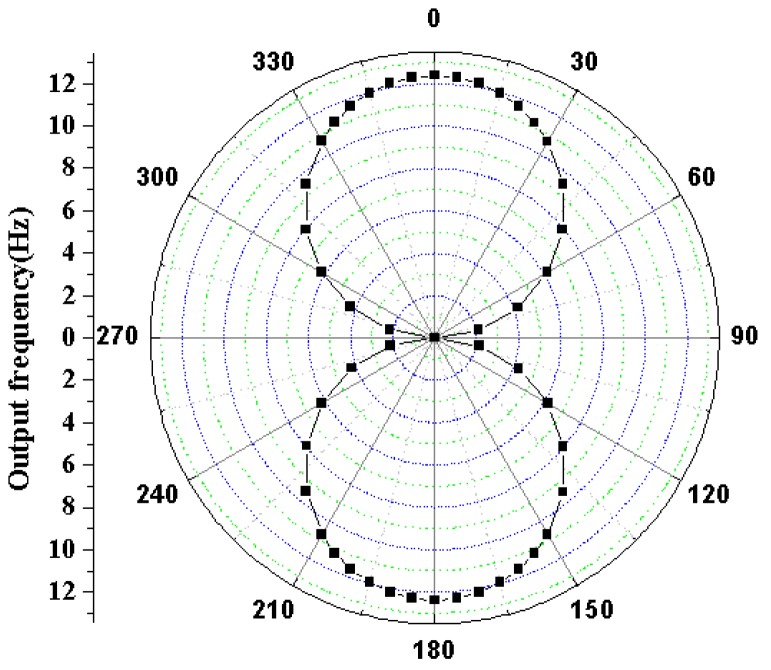
The simulation directivity of hair accelerometer using frequency output at an acceleration input of 1 g.

The simulation directivity of hair accelerometer using frequency output at an acceleration input of 1 g is shown in Figure 8.

With respect to the direction of the applied external acceleration, the hair accelerometer’s directivity is simulated by rotating it over 360° with steps of 10°. The simulation figures are in close agreement with the theoretical response for a so-called figure-of-eight. The simulation results indicate that the hair accelerometer has a maximum responsivity for both 0° and 180°.

**Figure 9:**
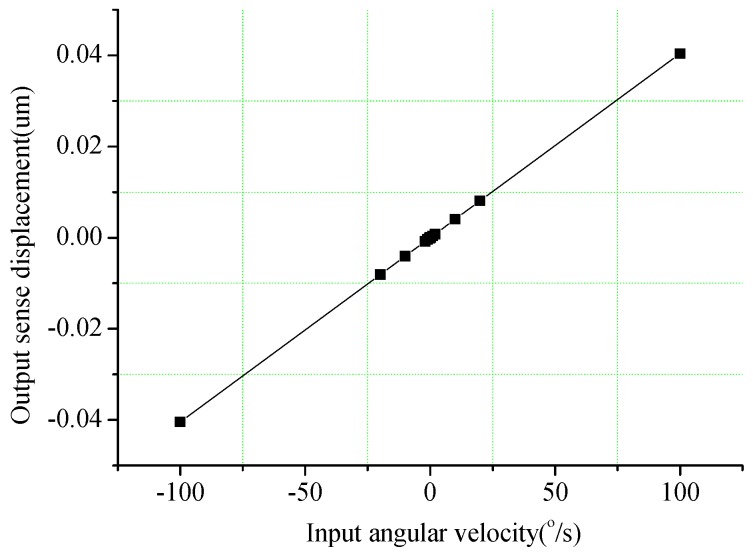
Output sense displacement in the angular velocity input along *y*-axis.

Finally the input-output characteristics of the angular velocity are analysed. The Coriolis forces from different angular velocity inputs are used to drive the sense mode of the gyroscope by the harmonic response simulation in the natural frequency of resonator mode. The resonant displacements of the gyroscope sense mode in different angular velocities are shown in Figure 9. The scale factor of the angular velocity detection shown in Figure 9 is 0.404 nm/deg/s. The new hair sensor can subsequently extract the angular velocity along the *y*-axis by the capacitance of sense combs.

**Figure 10:**
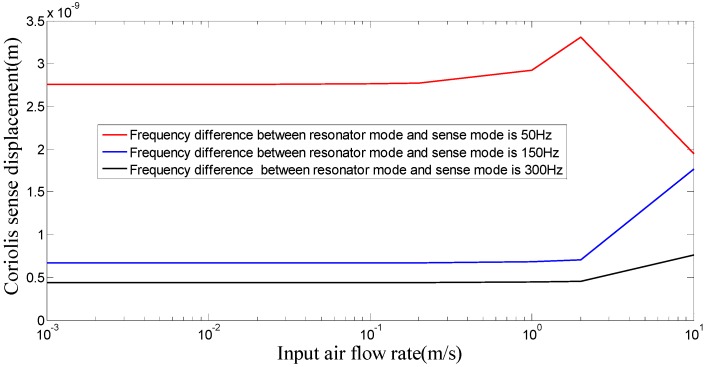
The influence of air flow rate on sense displacement of angular velocity.

In addition, the input of the acceleration or the air flow rate leads to the variation of resonator frequency, which will affect the signal detection of the angular velocity. The influence of the air flow rate on the sense displacement of angular velocity is shown in Figure 10 (the input angular velocity is 10°/s).

The decrease of the frequency differences between the resonator mode and the sense mode can increase the sensitivity of angular velocity sensing. However, a large angular velocity measurement error can be demonstrated due to the resonant frequency variation caused by the input of the air flow rate or the acceleration. Therefore, a certain frequency difference between the resonator mode and the sense mode must be maintained in order to suppress the error in displacement detection of the angular velocity, which is consistent with Equation (14).

### 4.2. Thermal Analysis

**Figure 11:**
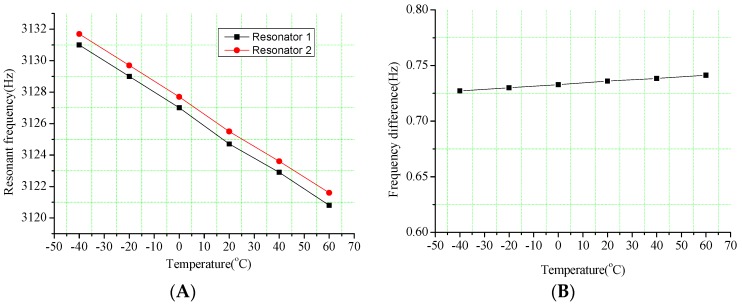
Frequency variations of resonators with temperature. (**A**) Frequency variations of resonators with temperature from −40 °C to 60 °C; (**B**) Frequency difference variations with temperature from −40 °C to 60 °C.

The resonators are utilized to convert the air flow and the acceleration into frequency signals in the new hair sensor. However, the natural frequencies of resonators are very sensitive to temperature variation. Since the Young’s modulus changes with temperatures, the frequency error of resonator brought by the temperature fluctuations is one of the major challenges. The frequency variations of resonators with temperature are shown in Figure 11. The apparent natural frequency drift of a single resonator is caused by the changes in temperatures from −40 °C to 60 °C. The simulation results demonstrate that a significant frequency drift rate of 0.102 Hz/°C shown in Figure 11A is validated in the single resonator. However, the frequency differences of a pair of resonators are remarkably reduced to 1.41 × 10^−4^ Hz/°C (that is 723 times optimization). Therefore, the frequency difference scheme can effectively eliminate the temperature influence on the measurement accuracy.

**Figure 12:**
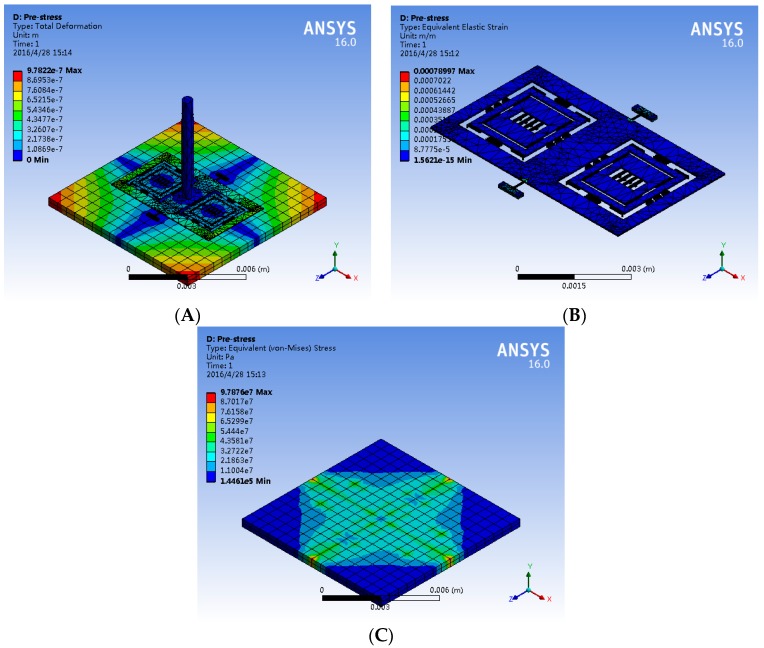
The deformation and stress distribution of hair sensor at 80 °C. (**A**) The deformation of the hair sensor; (**B**) The stress distribution of the silicon structures; (**C**) The stress distribution of the substrate.

Moreover, the substrate is used to match the expansion coefficient of the silicon structure layer as much as possible. However, there is a residual thermal stress between the structure layer due to temperature changes and the slight mismatch of the expansion coefficients. Accordingly, a simulation of the deformation and the stress distribution shown in Figure 12 is implemented at 80 °C in the hair sensor. The deformation results of the new hair sensor indicate that the maximum deformation of structure layer occurred in the end of the frame is 0.62 μm, and the maximum deformation of the substrate arisen in the border of substrate is 0.78 μm. Similarly, the stress distribution results demonstrate that the maximum stress of the structure, which does not reach the yield strength of 7 Gpa, presents at the root of the torsional beam with the value of 131 Mpa. The maximum stress in the counterpart substrate located at the bonding points is 97.8 Mpa (Figure 12C), which is within the yield strength. In summary, an excellent immunity to the temperature variation of the new hair sensor is demonstrated through the thermal analysis.

### 4.3. Air Flow Analysis

**Figure 13:**
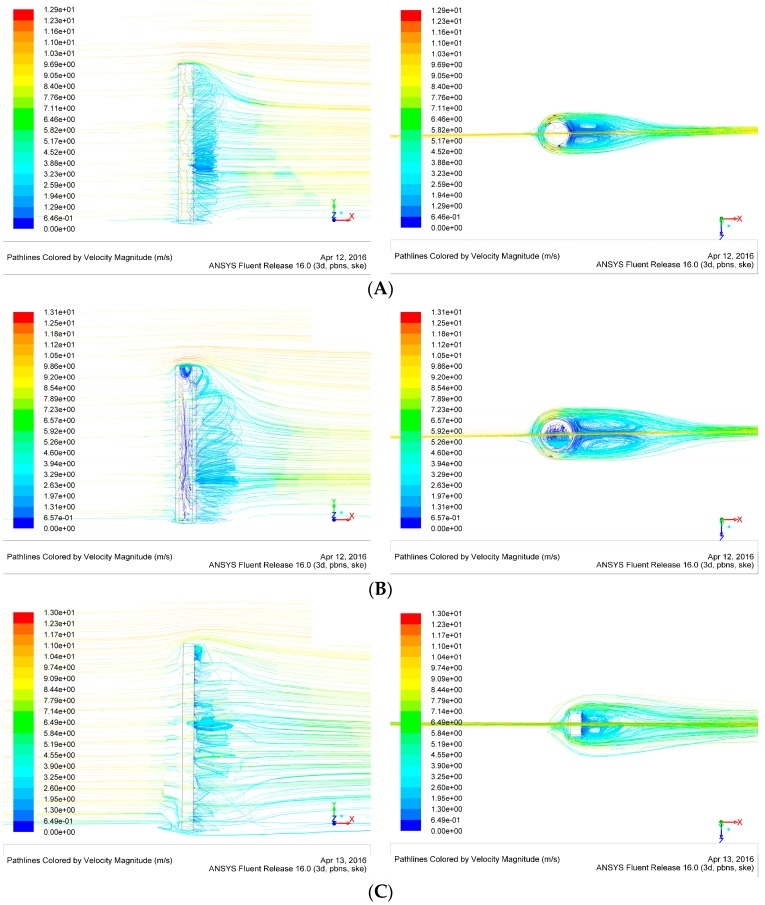
The air flow rate simulation of 10 m/s in different hair post shapes. (**A**) Circular hair post; (**B**) Hollow circular hair post; (**C**) Rectangle hair post.

The measurement of the air flow rate is converted into a frequency signal by the resonant transducer, which is similar to the detection of the acceleration signal. However, comparing to the inertial delivery methods of acceleration signals, the air flow rate is transferred by the surface pressure of the hair post before the resonant transformation. Figure 13 is the air flow rate simulation of 10 m/s in different hair post shapes. The simulation results indicate that the remarkable low velocity zone appears on the back of the hair post. Due to the flow velocity differences between the front and the back in the hair post, the pressure is generated on the hair surface, which is utilized to convert the air flow rate into a force. The low velocity zone in the hollow circular hair is much larger than that of the other two structures. Simultaneously, the increase in the surface area significantly extends the area of maximum pressure difference, which is beneficial for the efficiency improvement of the signal transmission.

## 5. Conclusions

In the paper, the design and the analysis of a new hair sensor are presented. The entire structure consists of a hair post, a torsional frame and a resonant signal transducer. The structural optimization of the hair sensor is implemented by the finite element method. The simulation results demonstrate that the hair sensor has a first mode of 240 Hz for the acceleration or the air flow sense, third and fourth modes of 3115 Hz for the resonant conversion, as well as fifth and the sixth modes of 3467 Hz for the angular velocity transformation, respectively. The input-output relationships are analyzed to verify the mechanical structural characteristics of the hair sensor. The analysis results illustrate simultaneous scale factors of 12.35 Hz/g for the acceleration sense, and 0.404 nm/deg/s for the angular velocity and a sensitivity of 1.075 Hz/(m/s)^2^ for the air flow. Moreover, the thermal analysis confirms the frequency difference scheme of resonant transducer can effectively eliminate the temperature influences on the measurement accuracy. Air flow analysis indicates that the increase in the surface area of hair post is significantly beneficial for efficiency improvement in the signal transmission. In summary, the structure of the new hair sensor is proved to be feasible by comprehensive simulation and analysis. As future work we plan to fabricate the hair sensor. Two closed-loop self-excitation circuits will be implemented to lock to the natural frequencies of two resonators, respectively. Two frequency detection circuits will be designed to measure the frequency of two self-excitation circuits. The capacitive readout circuit and subsequent conditioning circuit will be fullfilled to obtain the angular velocity. Finally, performance experiments of the hair sensor will be implemented to verify the differences between the theoretical analysis and test results.

## Figures and Tables

**Table 1:** Structure parameters.

Parameter		Value	Parameter	Value
Length × Width (µm)	Torsional beam	400 × 25	*B* (µm)	1850
	Drive suspension beam	440 × 10	*l*_h_ (µm)	3000
	Sense suspension beam	960 × 10	*d*_1_ (µm)	3
	Torsional frame	7000 × 4000	Bias voltage in coupled comb *V (V)*	12
	Outer frame	2100 × 2300	Torsional stiffness *k*_o_ (N·m/rad)	4.56 × 10^−5^
	Inner frame	1500 × 1700	Proof mass of resonator *m*_r_ (kg)	4.2 × 10^−^^7^
Diameter × Height (µm)	Hair post	600 × 6000	Proof mass of inner frame *m*_s_ (kg)	2.34 × 10^−7^
Coupled comb number *n*		242	Drive stiffness of resonator *k*_r_ (N/m)	152.62
Overlap length of coupled comb *L* (µm)		35	Sense mode stiffness *k*_x_ (N/m)	14.69
Structure thickness *H* (µm)		50	Sense mode Q-factor *Q_x_*	20
Moment of inertia *J* (kg·m^2^)		2 × 10^−11^	Q-factor of resonator *Q_z_*	200
Viscous damping coefficient *b* (N·m·s/rad)		3 × 10^−8^		

**Table 2:** Material properties of the hair structure.

Material	Silicon	Su-8 Photoresist (Hair Post)
Density (kg/m^3^)	2330	1200
Young’s modulus (N/m^2^)	1.7 × 10^11^	4.95 × 10^9^
Thermal conductivity (W/(m·K))	191	0.2

**Table 3:** The first 7th modes of the hair sensor.

Modal	1	2	3	4	5	6	7
Frequency (Hz)	240	2537	3115	3117	3467	3470	3746

**Table 4:** Performance comparison of three kinds of hair structure.

	Circular Hair Post	Hollow Circular Hair Post	Rectangle Hair Post
Structure Dimension (um)	600 × 6000	800 × 528 × 6000	752 × 376 × 6000
	diameter × height	outer diameter × Inner diameter × height	Length × width × height
Displacement sensitivity of acceleration (um/g)	1.587	1.591	1.591
Frequency sensitivity of acceleration (Hz/g)	12.35	12.37	12.38
Displacement sensitivity of air flow rate (um/(m/s)^2^)	0.138	0.187	0.171
Frequency sensitivity of air flow rate (Hz/(m/s)^2^)	1.075	1.458	1.333

**Table 5:** Performance comparison of two kinds of hair structure.

	I-Shape Hair Post	II-Shape Hair Post
Structure Dimension (um)	400 × 3000 (top) and 900 × 3000 (bottom)	900 × 3000 (top) and 380 × 3000 (bottom)
	(diameter × height)	(diameter × height)
Displacement sensitivity of acceleration (um/g)	1.600	2.042
Frequency sensitivity of acceleration (Hz/g)	12.45	15.89
Displacement sensitivity of air flow rate (um/(m/s)^2^)	0.140	0.139
Frequency sensitivity of air flow rate (Hz/(m/s)^2^)	1.09	1.08
